# Landslide susceptibility zonation using the analytical hierarchy process (AHP) in the Great Xi’an Region, China

**DOI:** 10.1038/s41598-024-53630-y

**Published:** 2024-02-05

**Authors:** Xiaokang Liu, Shuai Shao, Shengjun Shao

**Affiliations:** 1 Institute of Geo-Engineering, Xi’an University of Technology, P.O. Box:710048, Xi’an, China; 2 Shaanxi Provincial Key Laboratory of Loess Mechanics and Engineering, P.O. Box:710048, Xi’an, China; 3 Department of Architecture and Urban Planning, Xi’an University of Technology, P.O. Box:710048, Xi’an, China

**Keywords:** Environmental sciences, Natural hazards

## Abstract

This study aims to delineate landslide susceptibility maps using the Analytical Hierarchy Process (AHP) method for the Great Xi’an Region, China, which is a key planning project for urban construction in Shaanxi Province, China from 2021 to 2035. Multiple data as elevation, slope, aspect, curvature, river density, soil, lithology, and land use have been considered for delineating the landslide susceptibility maps. Spatially thematic layers and distributed maps of all the aforementioned parameters were created in a GIS environment. Determine the relative importance of these thematic layers in the occurrence of landslides in the study area concerning historical landslide data to assign appropriate weights. Landslide sensitivity maps were generated by a weighted combination in a GIS environment after being analyzed by the AHP method. The sensitivity maps were categorized as “very high (11.06%), high (19.41%), moderate (23.03%), low (28.70%), and very low (17.80%)”. Overlay analysis of the test data with the LSM showed that the moderate to very high landslide susceptibility zones were able to contain 82.58% of the historic landslides. The results of the study help determine the landslide-prone areas in the area and provide a reference for subsequent construction. In addition, the analysis of landslide susceptibility in the area contributes to the study of landslides in similar loess sites.

## Introduction

Over the years, cities around the world have grown rapidly, urban functions have extended to the surrounding areas, and metropolitan areas have emerged^1^. The excavation, filling, and change of the original land type in urban construction are the important causes of landslides^2–4^. A large number of landslides have occurred during urban expansion in China, especially in the west^5^, most of these landslides are concentrated in the Loess Plateau and have caused multiple disasters such as traffic disruptions, river blockages, farmland, factories, and housing burials^6^. The causes of landslides are various, the excavation and mechanical vibration in road network engineering, the foundation replacement of factories and houses, and the change of land use form make the original topography, groundwater circulation, and geological environment change. In addition, the cyclic load of vehicles, industrial water, and agricultural irrigation may also trigger landslides^7,8^. Therefore, it is necessary to analyze the risk of landslides in urban planning and construction projects.

Landslide sensitivity assessment is an important component of landslide risk analysis. Landslide susceptibility zoning (LSZ) is the process of determining the probability of a landslide occurring in an area and presenting the landslide risk area spatially in the form of a landslide susceptibility map (LSM)^9,10^. Landslide susceptibility areas are usually delineated based on geologic activities, soil structure, topography, hydrologic environment, and human activities as landslide causative factors and combined with GIS techniques^11^. A general overview of different LSZ techniques applied has been broadly categorized as physical methods, heuristic methods, and statistical methods^12^. Heuristic methods are based on a priori knowledge to predict landslides by assuming subjective weights of different factors that may lead to the occurrence of landslides^13^. Physical methods predict landslides through a rigorous mathematical process based on the physical mechanisms by which landslides occur^9^, such as limit equilibrium, finite element, and limit analysis methods. Although physical methods provide a more realistic response to the mechanism and process of landslide occurrence, the use of such methods requires precise mechanical parameters and therefore can only be applied to a certain type of slope stability calculation^14^. The statistical approach is based on analyzing and predicting historical landslide data in the study area^15^. Thanks to the development of machine learning algorithms, this method has an absolute advantage in landslide susceptibility prediction research, such as LR^16,17^, SVM^18^, ANN^19^, RF^20^, etc. Machine learning based on statistical methods has a powerful data processing ability and does not require strict display functions or a large amount of a priori knowledge^21^, but it can lead to incorrect results when insufficient data or insufficient accuracy is available, so sufficient and accurate historical landslide data is the basis for using this method^22^.

The Analytic Hierarchy Process (AHP) is a typical heuristic model that combines qualitative and quantitative methods to decompose a complex decision problem into different levels. This approach quantifies and translates opinions into coherent decision models^23,24^. The greatest advantage of the hierarchical analysis method over the currently popular machine learning methods is that it does not require the knowledge of any a priori relationship between historical landslide data or landslide genesis, and the method controls the uncertainty of subjective judgment through the consistency ratio rule^25,26^. Therefore, using this method for landslide susceptibility zoning of the study area can yield satisfactory results when landslide historical data are too scarce or their accuracy is questionable. In recent years, even with the rapid development of machine learning techniques, hierarchical analysis is still widely used in the study of landslide susceptibility, e.g., Abay et al. (2019) used the method to evaluate landslide susceptibility in the Tarmaber area of Ethiopia^27^; Sonker et al. (2021) combined the GIS method to map landslide susceptibility in the Sikkim-Himalayan region of India^28^; and Panchal & Shrivastava (2022) used the AHP method for the slope of National Highway 5 in India to Landslide hazard evaluation was carried out^29^; Das et al. (2022) carried out landslide susceptibility zoning for the Darjeeling Himalayan region^25^; He et al. (2019) used hierarchical analysis of hierarchies (AHP) to assess landslide susceptibility in southern Anhui, China^30^; Zhou et al. (2023) used the method to evaluate landslide susceptibility in Yunxian County, southwestern China for PV power farm construction^31^; El Jazouli et al. (2019) used the method for landslide susceptibility mapping in Oum Er Rbia high basin (Morocco)^32^; Zangmen et al. (2023) used it for landslide susceptibility zoning in Bafoussam-Dschang region (West Cameroon)^33^; Nguyen et al. (2023) evaluated the effect of rainfall on landslide susceptibility in the mountainous region of central Vietnam using the AHP method^34^; Kohno et al. (2023) used the Analytic Hierarchy Process (AHP) method for landslide susceptibility mapping of the entire slopes of the Japanese archipelago, and performed a GIS analysis^35^.

The Greater Xi’an area is a major planning project of the local government for the period of 2021–2035, and the prevention and control of geologic hazards is an important part of its research. In this study, we collected historical landslide samples from the planning area, extracted landslide-inducing factor data through DEM and various thematic maps, and used hierarchical analysis to integrate landslide occurrence parameters into the GIS environment to map landslide-prone areas in the region. The final research results can provide support for the later construction of the area.

## Materials and methods

### Study area

**Figure 1:**
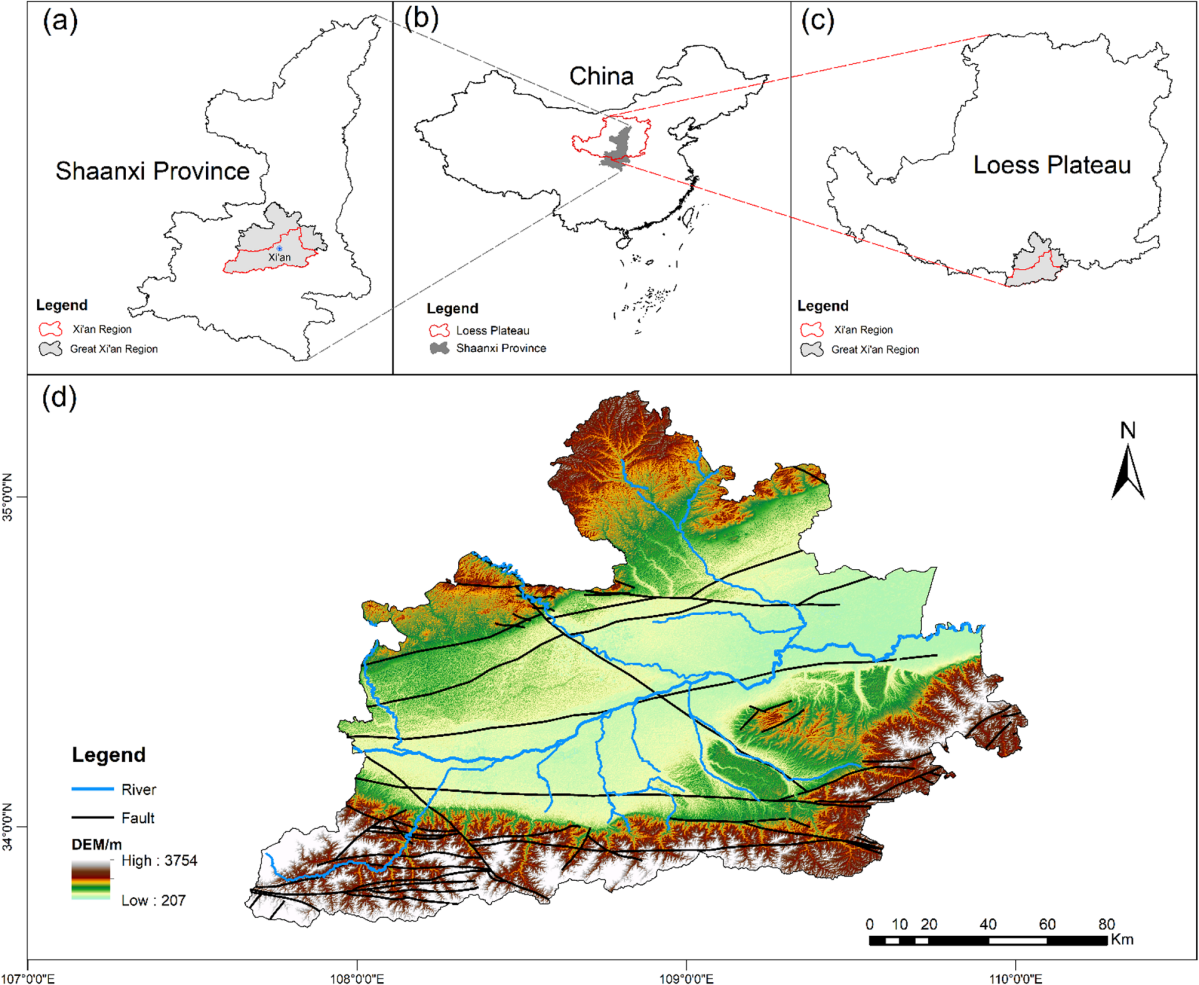
Study area map: (**a**) Location of the study area in Shaanxi Province, China, (**b**) the map of China, (**c**) Location of the study area on the Loess Plateau, (**d**) Study area information map.

The study area is located in the central part of Shaanxi Province, China (Fig. 1a and b), and belongs to the southern Loess Plateau (Fig. 1b and c), with a total area of about 20,600 square kilometers. Figure 1d shows the information on the study area. The overall terrain is high in the north and south, low in the middle, high in the west and low in the east, and gently inclined to the east. The altitude is between 207 and 3757 m. Affected by the rise of the earth’s crust, floods, erosion of the Weihe River and its tributaries, and loess accumulation, the landform types are diverse, mainly including alluvial plain, diluvial plain, piedmont alluvial-pluvial plain, loess tableland, and bedrock mountain. The south is the Qinling fold belt and the north is the Ordos block. From the old to the new, there are Eocene and Oligocene of Paleogene, Miocene, and Pliocene of Neogene, Lower Pleistocene, Middle Pleistocene, Upper Pleistocene and Holocene of Quaternary. The Quaternary is widely distributed in the Great Xi’an Region. The lithology is mainly silty clay, loess, sand, and gravel. The genesis is complex, mainly formed by alluvial, diluvial, and aeolian deposits. The maximum thickness of Quaternary in the area is about 800 m, the river terrace is generally more than 400 m, and the thickness of loess tableland is generally less than 300 m. The climate condition is semi-arid and semi-humid continental monsoon climate. The annual average temperature is 13–15°, and the annual average rainfall is 537.5–1028.4 mm. It decreases from south to north, mainly from July to September.

### Methods

**Figure 2:**
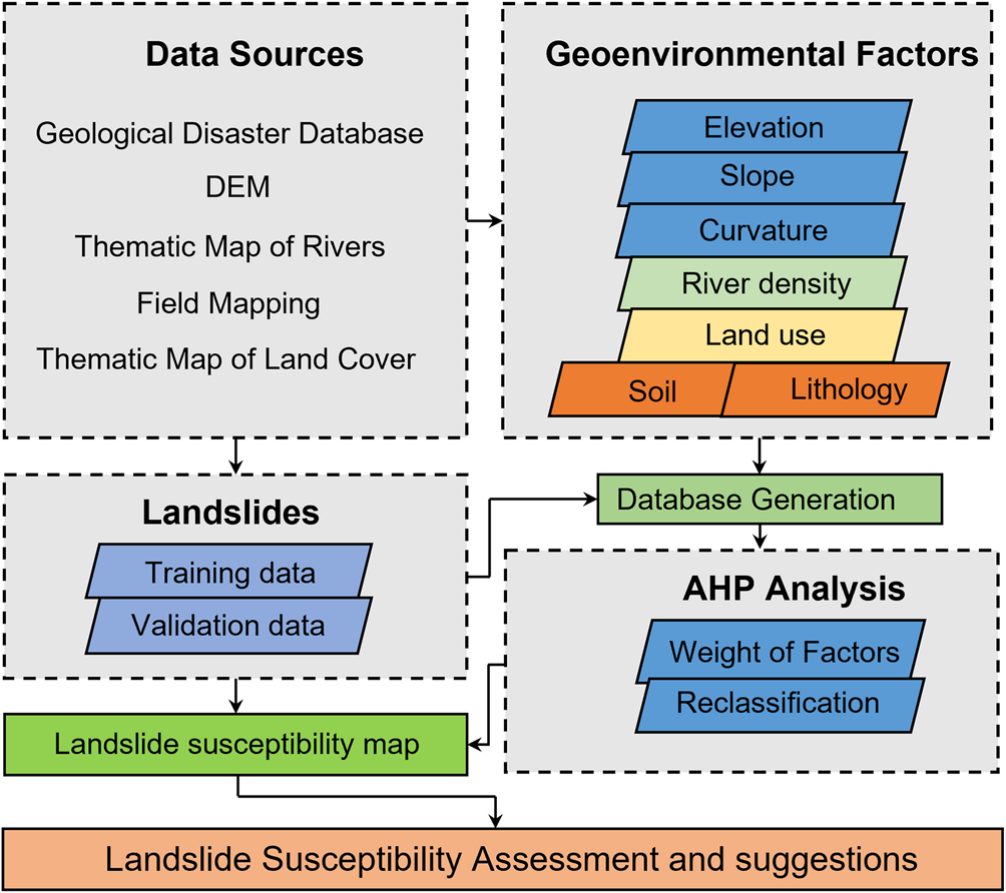
The framework of methods.

The method flow used in this study is shown in Fig. 2, which mainly includes data collection, factor determination, generation of raster database, AHP analysis, and drawing landslide susceptibility map. Finally, the landslide susceptibility assessment of the area is carried out and the prevention and control suggestions are given.

#### Data collection

Table 1 shows the data sources and data formats of this study. A total of 1075 slope disaster points were obtained from the Resource and Environment Science and Data Center (www.resdc.cn) geological disaster database, including 506 collapse points, 534 landslide points, and 35 debris flow points (collectively referred to as landslide in this study). Among them, 70% were used for model calculation and 30% for model verification. In addition, slope, aspect, and profile curvature were extracted from the digital elevation data using the spatial analysis toolbox of ArcGIS software, and stream density was derived through the Line Density Calculator tool in Arcgis10.8.

**Table 1:** Data information of this study.

Data type	Type of data or resolution	Source
Digital Elevation Model	Raster (30 m)	ASTER GDEM V3 (www.gscloud.cn)
Land use and Land cover	Raster (30 m)	National Catalogue Service for Geographic Information, China (www.webmap.cn)
Geological map	Shap (1:250,000)	Resource and Environment Science and Data Center, China (www.resdc.cn)
River network map	Shap (1:250,000)	National Catalogue Service for Geographic Information, China (www.webmap.cn)

#### Definition of conditioning factors

The triggering factors of landslides are defined according to the relevant literature and the characteristics of the study area. Since this study aims to provide a reference for risk identification and prevention in the future construction of the Greater Xi’an Region, we should take into account the easy access to data when determining factors, that is, to avoid the actual data acquisition being too complicated or costly. Firstly, based on the basic terrain conditions, this paper selects altitude, slope, aspect, and profile curvature as the basic landslide triggering factors. Elevation influences the type of regional vegetation, intensity of human activities, and water accumulation area. It has an indirect correlation with landslides and is a commonly used factor in landslide sensitivity evaluation^36,37^. The slope gradient affects the distribution of the stress field inside the clivus. Theoretically, the stress at the toe of the clivus is positively correlated with the magnitude of the slope, and the greater the stress at the toe of the clivus, the greater the possibility of slope instability^37^. In addition, the slope gradient also affects the infiltration process^38^. Therefore, slope gradient is considered to be an important topographic factor that directly affects landslides^39^. Different aspects have different levels of solar energy absorption, rainfall infiltration and runoff, and surface fragmentation, which indirectly affect slope stability^37,40^. Curvature influences landslide occurrence by controlling the erosion process and surface runoff from the slope^41^. Secondly, because the study area is located in the Loess Plateau, river erosion, infiltration, and human activities have a great influence on the stability of the loess slope, so river density and land use are also selected as triggering factors.

The optimal resolution for describing landslide condition factors varies, and there is currently no standardized spatial resolution for landslide susceptibility modeling^42^. In previous studies, a grid cell resolution of 30 m was commonly used^22,43^, which can not only effectively represent topographic features but also avoid excessive calculations^44^. Therefore, all the landslide condition factors in this paper also use 30 m resolution.

The original continuous type cannot be assigned weight values directly, so the natural breaks method is used for classification because this method can minimize the differences within each subclass and maximize the differences between neighboring subclasses, thus avoiding human subjective interference^45,46^. By reclassifying the extracted data, the elevation can be divided into three categories: low (207–809 m), moderate (809–1528 m) and high (1528–3754 m). The slope is divided into six categories: 1 (< 5°), 2 (5°–10°), 3 (10°–20°), 4 (20°–30°), 5 (30°–40°), 6 (> 40°). The aspect classification is: plane (< 0°), north (0–22.5° and 337.5°–360°), northeast (22.5–67.5°), east (67.5–112.5°), southeast (112.5–157.5°), south (157.5–202.5°), southwest (202.5–247.5°), west (247.5–292.5°), northwest (292.5–337.5°). Curvature is divided into concave, linear, and convex three categories.

Seven land use types were obtained through land cover data: Cultivated Land, Forest, Grassland, Shrubland, Wetland, Water Bodies, and artificial surfaces.

Geological maps can be used to extract lithology and soil. The lithology of the study area is divided into five categories: igneous rock, metamorphic rock, consolidated sedimentary rock, eolian rock, fluvial rock, and weathered residuum. According to the soil classification method of the Harmonized World Soil Database (HWSD), the soil in the study area was divided into anthrosols, cambisols, fluvisols, luvisols, and gleysols.

The river density in the study area can be calculated by using the Arcgis10.8 linear density analysis tool and be divided into four categories by natural discontinuity method: 0–34.51, 34.51–57.14, 57.14–81.47, 81.47–144.26.

#### Analytical hierarchical process (AHP)

AHP is a mathematical method used for multi-criteria decision analysis. The characteristics of this method are that based on in-depth research on the nature, influencing factors, and internal relations of complex decision problems, it uses less quantitative information to make the decision-making thinking process mathematical, to provide a simple decision-making method for complex decision-making problems with multiple objectives, multiple criteria or unstructured characteristics^47–49^. This method can be used for quantitative analysis of triggering factors of geological disasters^33,50^. In this study, the program using the analytic hierarchy process is divided into three continuous steps: (1) Create the pair-wise matrix for each landslide trigger factor, (2) Calculate the weight of each factor, (3) Check the accuracy of the results by consistency ratio (CR; Saaty, 1977).

The classification of each trigger factor is compared in pairs, and the pair-wise matrix is established as follows:

1 $$ M = \left[ {\begin{array}{*{20}c} 1 & {{\text{a}}_{12} } & {a_{13} } & \ldots & {a_{1n} } \\ {a_{21} } & 1 & {a_{23} } & \ldots & {a_{2n} } \\ {a_{31} } & {a_{32} } & 1 & \ldots & \ldots \\ \vdots & \vdots & \vdots & \vdots & \vdots \\ {a_{n1} } & {a_{n2} } & \ldots & \ldots & 1 \\ \end{array} } \right] $$

2 $$ {\text{a}}_{ij} = \frac{{\text{weight of attribute i}}}{{\text{weight of attribute j}}} $$

where *a*_*ij*_ represents the comparison result of the *i*th factor relative to the *j*th factor. The range of values is 1–9^51^, which is determined by the rules of Table 2.

**Table 2:** Preference scale between two parameters in AHP.

Importance scale	Definition
1	Equal importance
3	Moderate importance
5	Strong importance
7	Very strong importance
9	Extreme importance
2,4,6,8	Intermediate values between two adjacent decisions
Reciprocals	Used for inverse comparison

The consistency of the results can be judged by the consistency ratio (CR), which is calculated by Eq. (3). A reasonable CR level is equal to or less than 0.1; otherwise, the pairwise matrix needs to be revised^51^.

3 $$ CR = \frac{{\lambda_{\max } - n}}{n - 1} \cdot \frac{1}{RI} $$

where λ_max_ is the maximum eigenvalue of the matrix, *n* is the maximum number of factors, *RI* is the random consistency index^51^, and the value criterion in Table 3.

**Table 3:** Average random consistency index (RI).

*n*	1	2	3	4	5	6	7	8	9	10	11
*RI*	0	0	0.58	0.90	1.12	1.24	1.32	1.41	1.45	1.49	1.51

To produce the landslide susceptibility map of the study area, factors such as elevation, slope, aspect, profile curvature, lithology, soil, land use, and river density were considered, the judgment matrix of RC < 0.1 was obtained, and the weight value of each factor was calculated. Then, using Arcgis10.8 software, the landslide susceptibility grid map is drawn and the susceptibility map is divided into five levels by the weight value, namely very high susceptibility areas, high susceptibility areas, moderate susceptibility areas, low susceptibility areas, and very low susceptibility areas, The five levels of susceptibility were determined by the natural breakpoint method, which is a widely recognized method for susceptibility level classification in landslide susceptibility map^52^.

## Results

### Landslide conditioning factors

**Figure 3:**
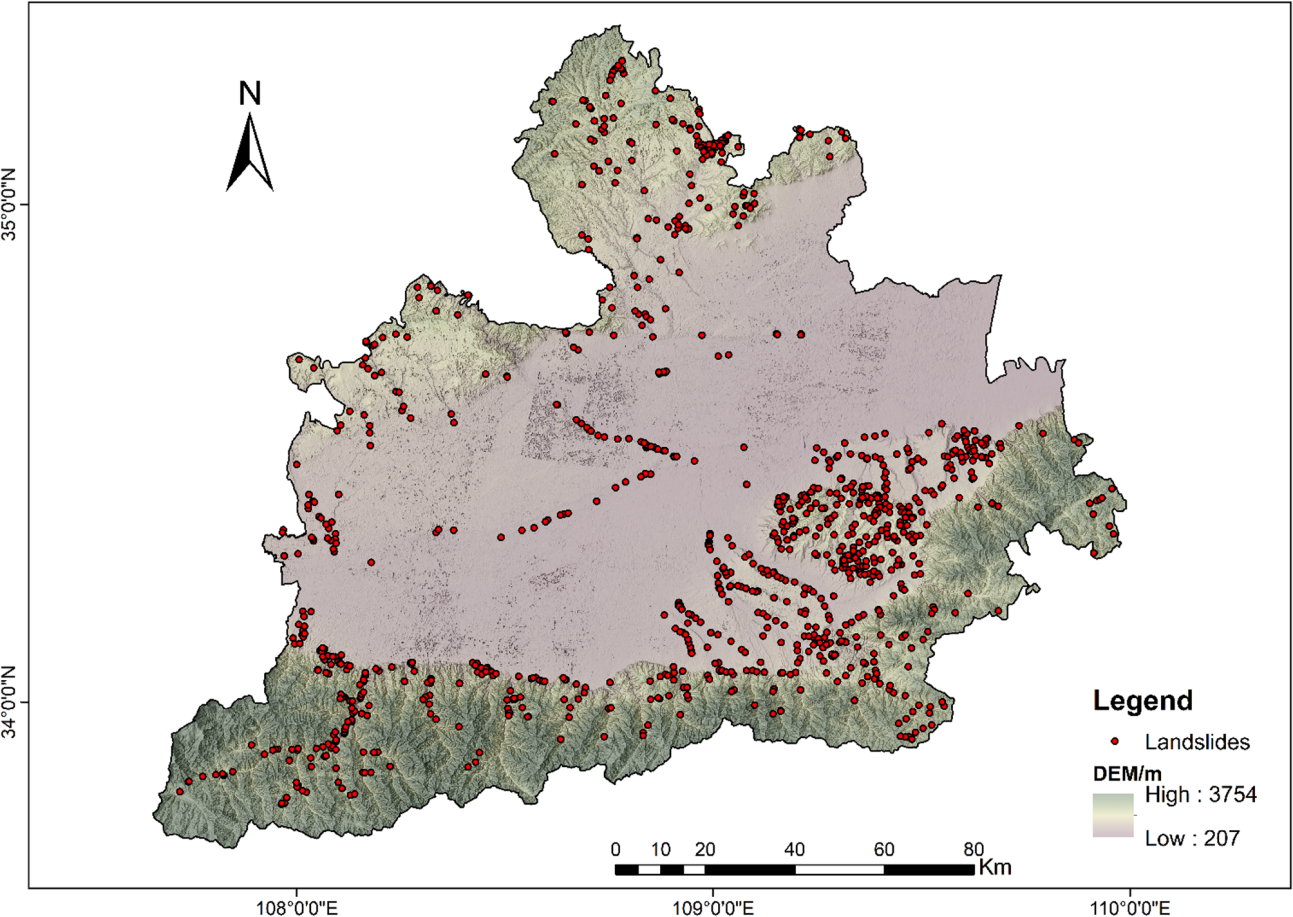
The distribution of landslides in the study area.

From the general distribution of landslide points (Fig. 3), the triggering factors seem to be closely related to morphology and human factors. Morphologically, they are located in the gully and the edge of the tableland. Among the human factors, urban construction land, agricultural land, and traffic construction are closely related. In addition, according to the study of loess landslides, rivers, and other water effects also have a great role in promoting landslides.

**Figure 4:**
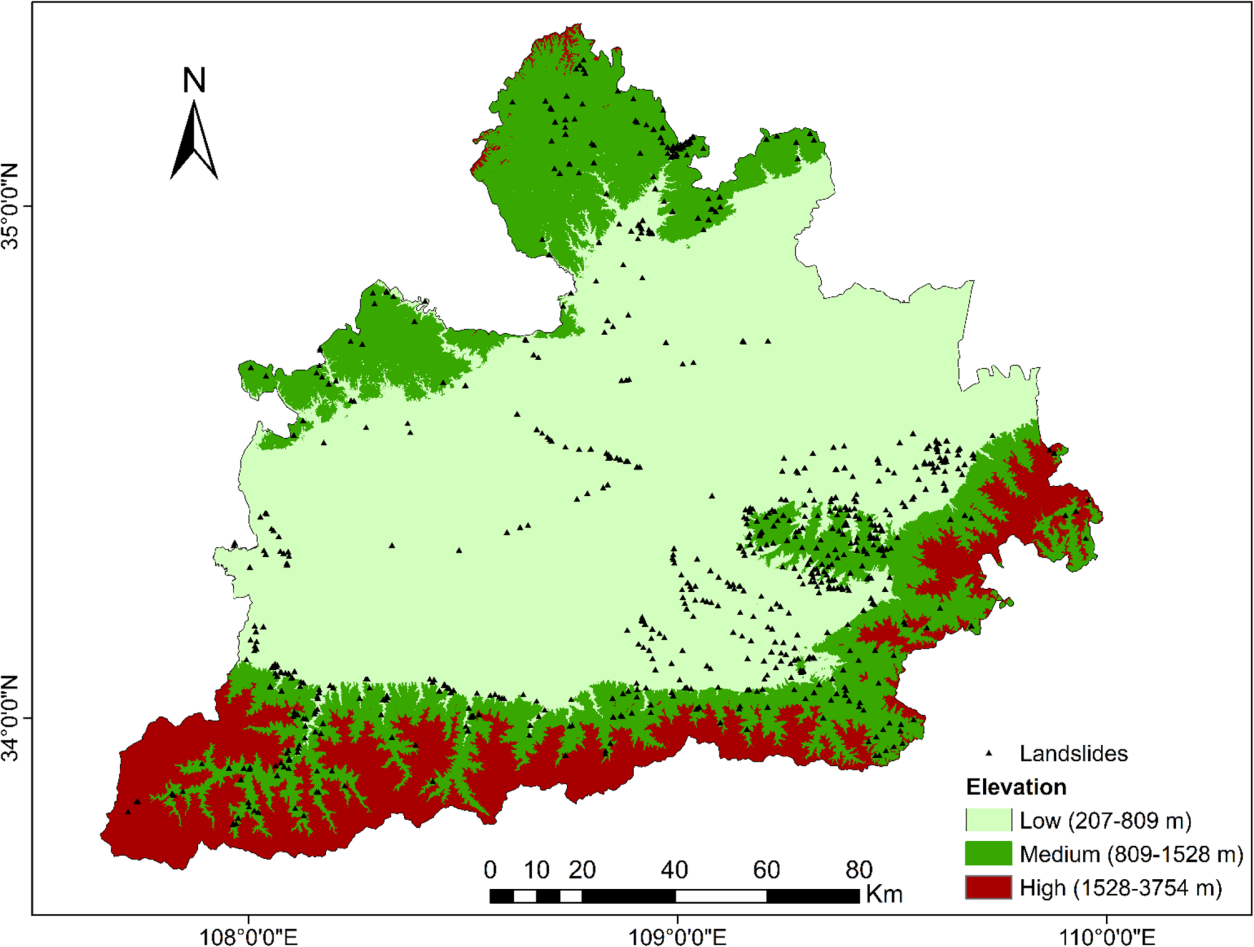
Interdependence of the Great Xi’an Region landslides with the elevation map.

**Figure 5:**
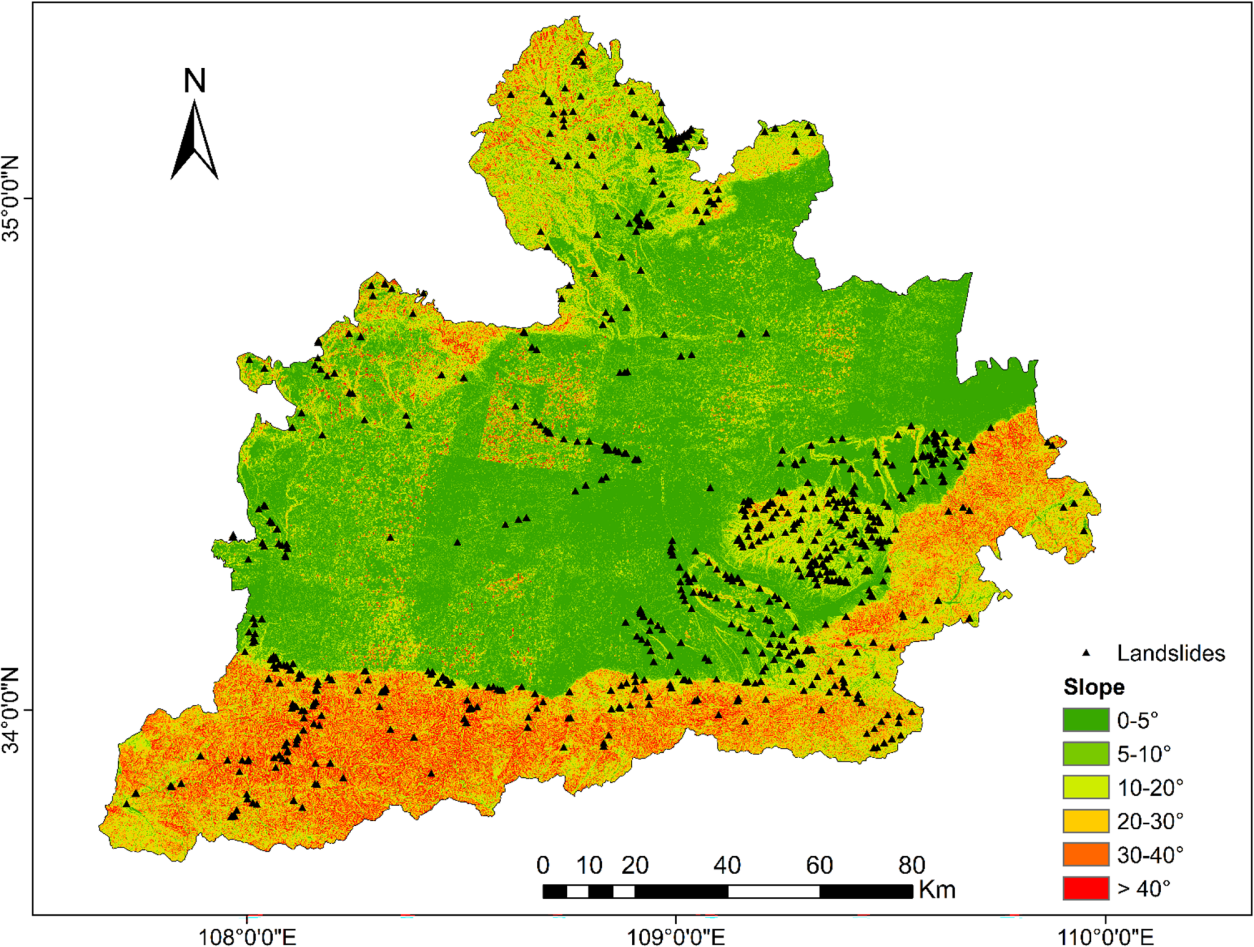
Interdependence of the Great Xi’an Region landslides with the slope map.

**Figure 6:**
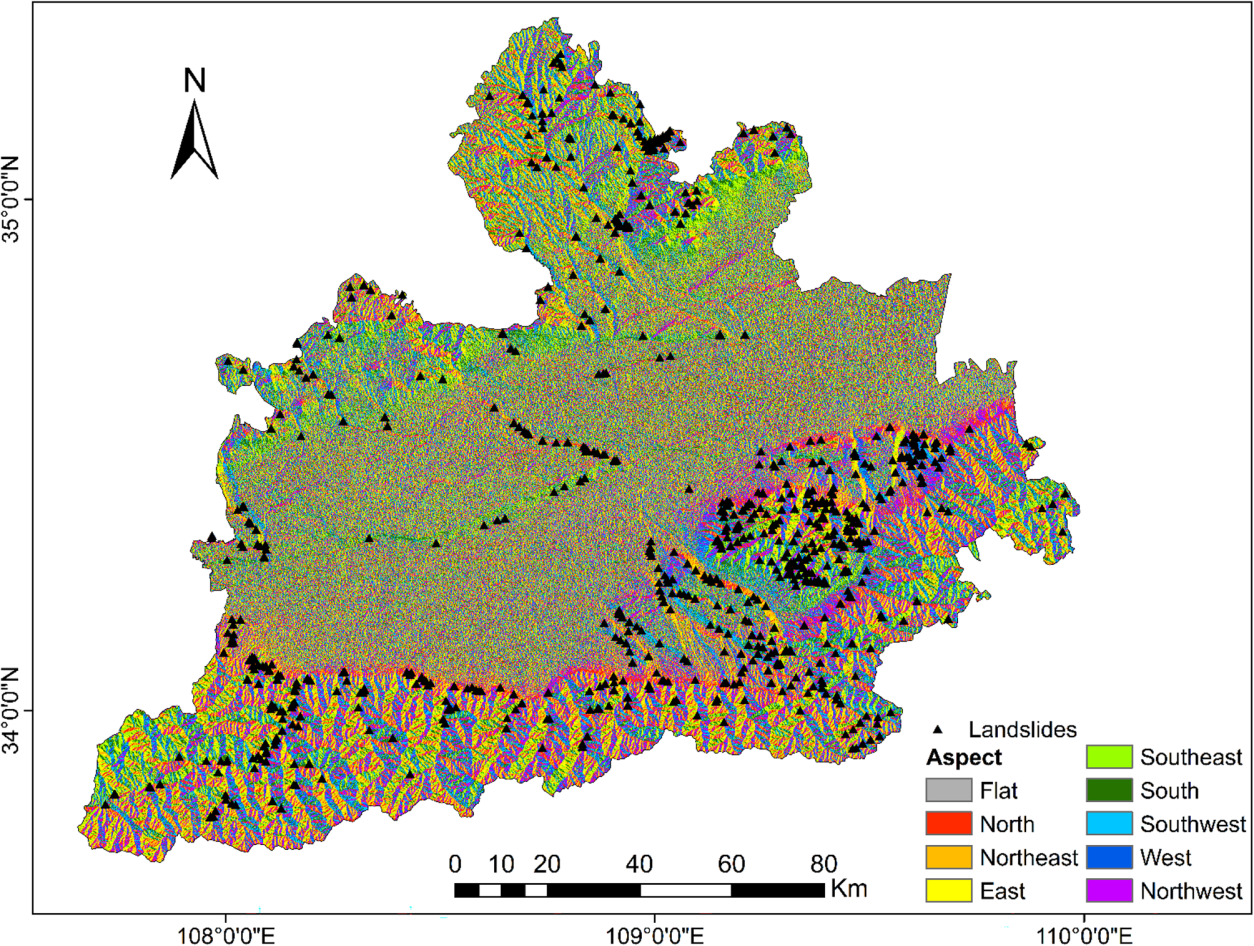
Interdependence of the Great Xi’an Region landslides with the aspect map.

**Figure 7:**
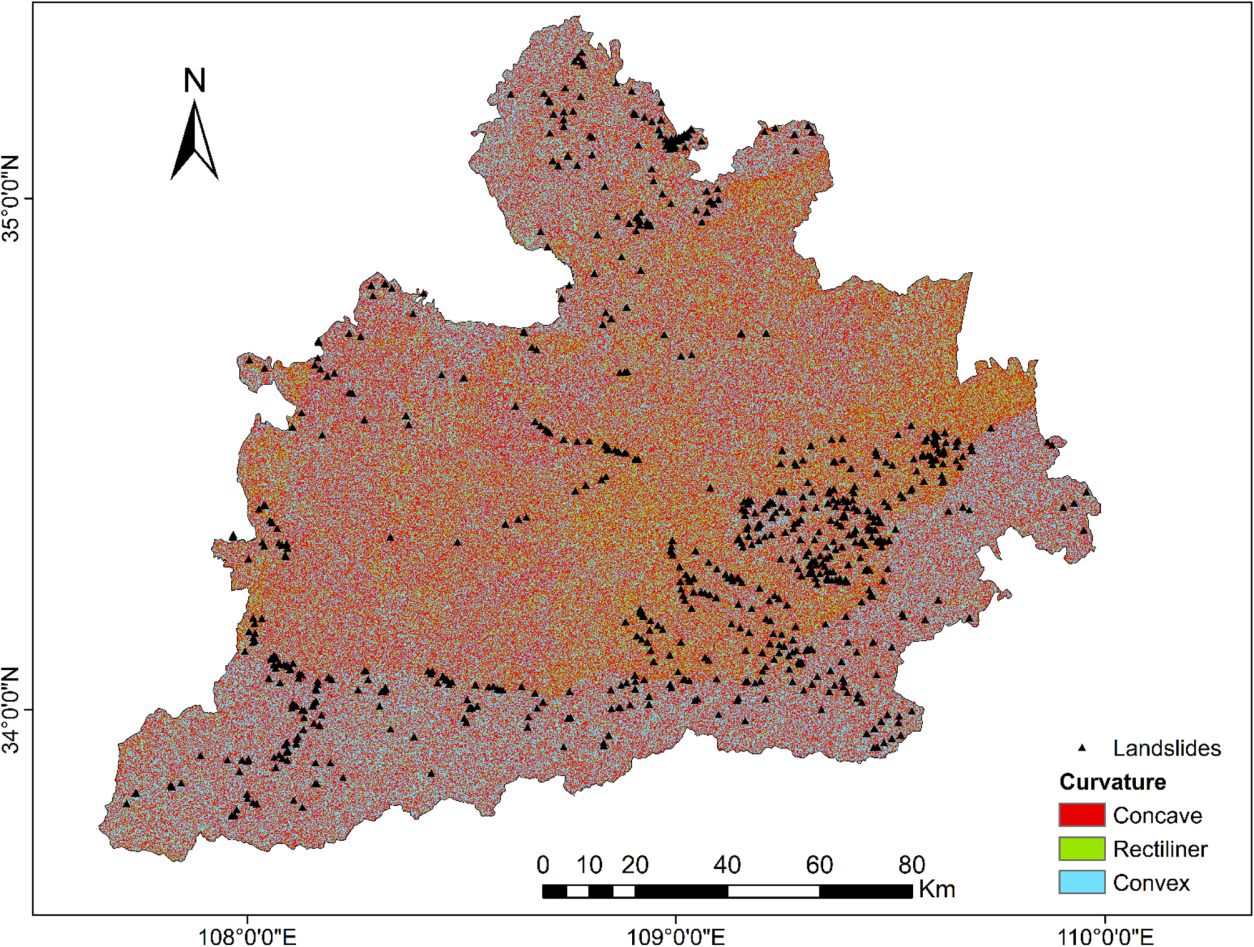
Interdependence of the Great Xi’an Region landslides with the curvature map.

**Figure 8:**
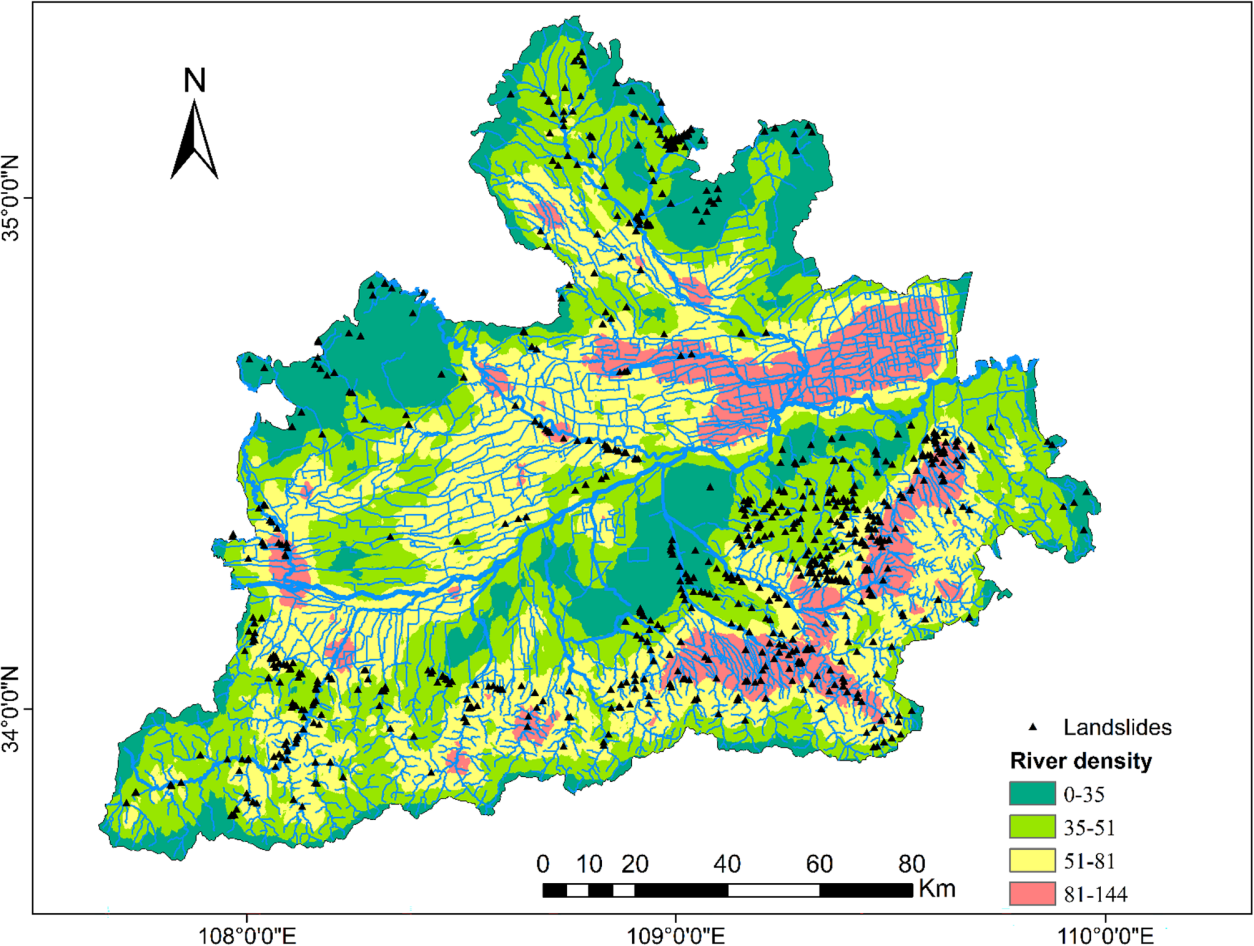
Interdependence of the Great Xi’an Region landslides with the river density map.

**Figure 9:**
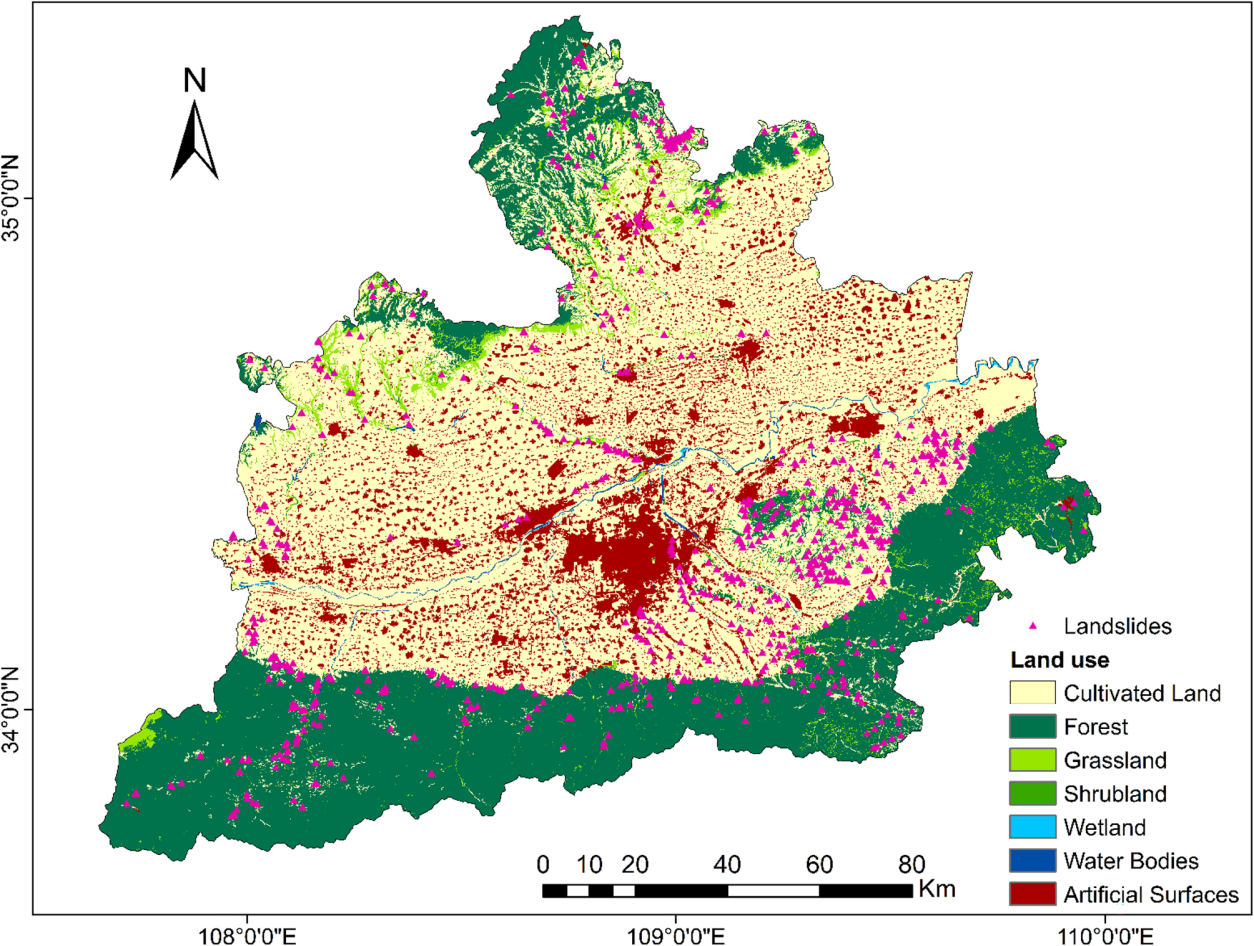
Interdependence of the Great Xi’an Region landslides with the land use map.

To be able to generate a comprehensive landslide susceptibility map in the Greater Xi’an Region, this paper associates the relevant thematic map with 1075 data in the landslide database, including 753 training sets and 322 validation levels (Fig. 3). Thematic maps are generated by selected different trigger factors. Slope, lithology, soil, river density, profile curvature, elevation, and aspect were selected as trigger factors to calculate their weight values and draw thematic maps (Tables 4, 5, Figs. 4, 5, 6, 7, 8, 9).

**Table 4:** Landslide factors used in susceptibility mapping and their parameters.

Factors	Classes	Training data (%)	Degree of preference	Weight	CR
Elevation	207–809	36	Moderate	0.283	0.057
	809–1528	61	High	0.643	
	1528–3754	3	Low	0.074	
Slope	0–5	6	Low	0.035	0.034
	5–10	19	High	0.151	
	10–20	34	Very high	0.444	
	20–30	16	High	0.151	
	30–40	13	High	0.151	
	> 40	12	Moderate	0.068	
Aspect	Flat	3	Low	0.025	0.003
	North	13	High	0.132	
	Northeast	13	High	0.132	
	East	13	High	0.132	
	Southeast	11	High	0.132	
	South	11	High	0.132	
	Southwest	14	High	0.132	
	West	12	High	0.132	
	Northwest	8	Moderate	0.048	
Curvature	Concave	32	Moderate	0.283	0.030
	Rectilinear	31	Low	0.074	
	Convex	37	Very high	0.643	
River density	0–35	18	Low	0.057	0.044
	35–51	27	High	0.263	
	51–81	24	Moderate	0.122	
	81–144	31	Very high	0.558	
Land use	Cultivated Land	49	High	0.270	0.075
	Forest	24	Moderate	0.115	
	Grassland	10	Low	0.059	
	Shrubland	0	Low	0.040	
	Wetland	0	Low	0.032	
	Water Bodies	0	Low	0.032	
	Artificial Surfaces	18	Very high	0.452	
Lithology	Igneous rock	6	Low	0.039	0.060
	Clastic sediments	14	High	0.203	
	Limestone	4	Moderate	0.087	
	Eolian(loess)	65	Very high	0.478	
	Fluvial	9	Moderate	0.115	
	Weathered residuum	2	Moderate	0.079	
Soil	Anthrosols	35	High	0.192	0.080
	Cambisols	43	Very high	0.450	
	Fluvisols	9	Moderate	0.097	
	Luvisols	2	Low	0.040	
	Regosols	10	High	0.220

**Table 5:** Normalized pair-wise comparison and weight values of landslide attributes of the AHP model.

	AS	EL	PC	RD	LU	SO	LI	SL	NW	CR
AS	1	1/5	1/3	1/3	1/3	1/3	1/3	1/7	0.03	0.050
EL	5	1	3	2	3	3	3	1/3	0.20	
PC	3	1/3	1	1/3	2	2	2	1/3	0.10	
RD	3	1/2	3	1	1/2	2	2	1/3	0.12	
LU	3	1/3	1/2	2	1	2	2	1/3	0.11	
SO	3	1/3	1/2	1/2	1/2	1	1	1/4	0.07	
LI	3	1/3	1/2	1/2	1/2	1	1	1/4	0.06	
SL	7	3	3	3	3	4	4	1	0.35	

*AS* aspect, *EL* elevation, *PC* profile curvature, *RD* river density, *LU* land use, *SO* soils, *LI* lithology, *SL* slope, *NW* normalized weight.

The elevation is divided into low altitude (207–809 m), moderate altitude (809–1528 m), and high altitude (1528–3754 m), which correspond to the middle (36%), high (31%) and low (3%) areas of landslide, respectively (Table 4, Fig. 4).

The slope is divided into 6 categories: 1(< 5°), 2(5–10°), 3(10–20°), 4(20–30°), 5(30–40°), 6(> 40°). These grades correspond to low (6%), high (19%), very high (34%), high (16%), high (13%), and Moderate (12%) landslide susceptibility (Table 4). High landslide susceptibility accounted for 48% (class 2,3 and 5) and very high susceptibility accounted for 34% (class 4). Therefore, the slope of 5–50° is a high incidence area of landslide (Fig. 5).

According to Table 4 and Fig. 6, it can be seen that the distribution of landslides on the plane type is very small (3%), and the distribution of other slopes is relatively uniform. The northwest direction is defined as Moderate (8%), and others are defined as high (11–14%).

Table 4 and Fig. 7 show the concave (32%), straight (31%), and convex (37%) profile curvature categories. Concave and convex represent 69% of landslides. Among them, the straight or rectilinear is mainly distributed in the central part of the study area.

Considering all rivers in the study area (including natural rivers and artificial rivers), the calculated density is between 0 and 144, which is divided into four categories (Table 4): 0–35,35–51,51–81,81–144, corresponding to low (18%), high (27%), Moderate (24%), very high (31%) landslide rates. Figure 8 shows the distribution of landslides and rivers. It can be seen that a large number of landslides are distributed along the river.

Table 4 and Fig. 9 show the distribution of landslides on different land use types: Cultivated Land (49%), Forest (24%), Grassland (10%), Shrubland (0%), Wetland (0%), Water Bodies (0%), Artifical Surfaces (18%). It can be seen from Fig. 9 that landslides are mainly concentrated in or near the classification related to human activities, and the landslides counted on Forest and Grassland are also close to Cultivated Land or artificial surfaces.

**Figure 10:**
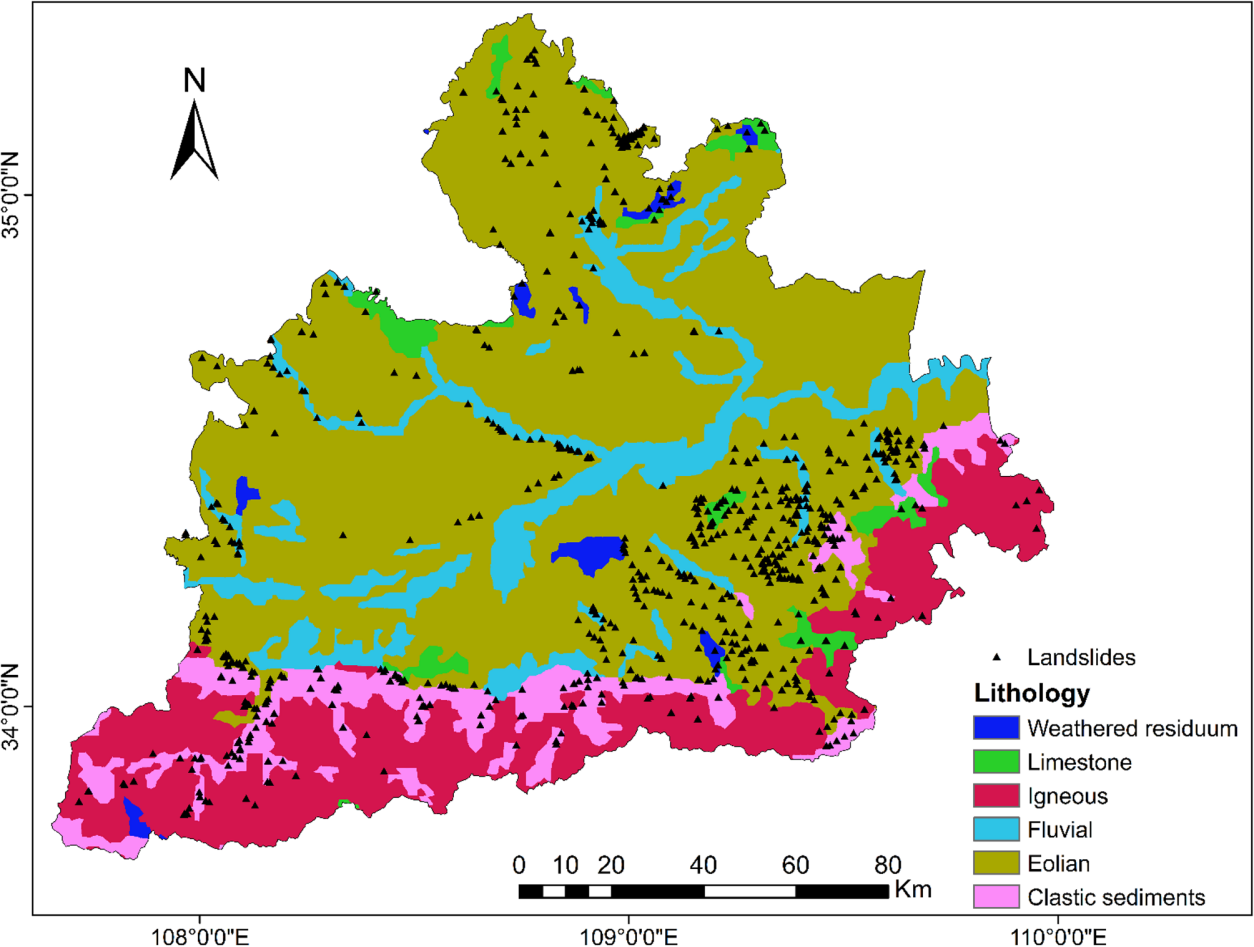
Interdependence of the Great Xi’an Region landslides with the lithology map.

The lithology classification of landslides in the study area is shown in Table 4 and Fig. 10: igneous rock (6%), clastic sediments (14%), limestone (4%), eolian rock (65%), fluvial rock (9%), weathered outcrop (2%). Among them, eolian rock (mainly loess) accounts for 65% of the total landslide, that is to say, the loess landslide in the study area is representative.

**Figure 11:**
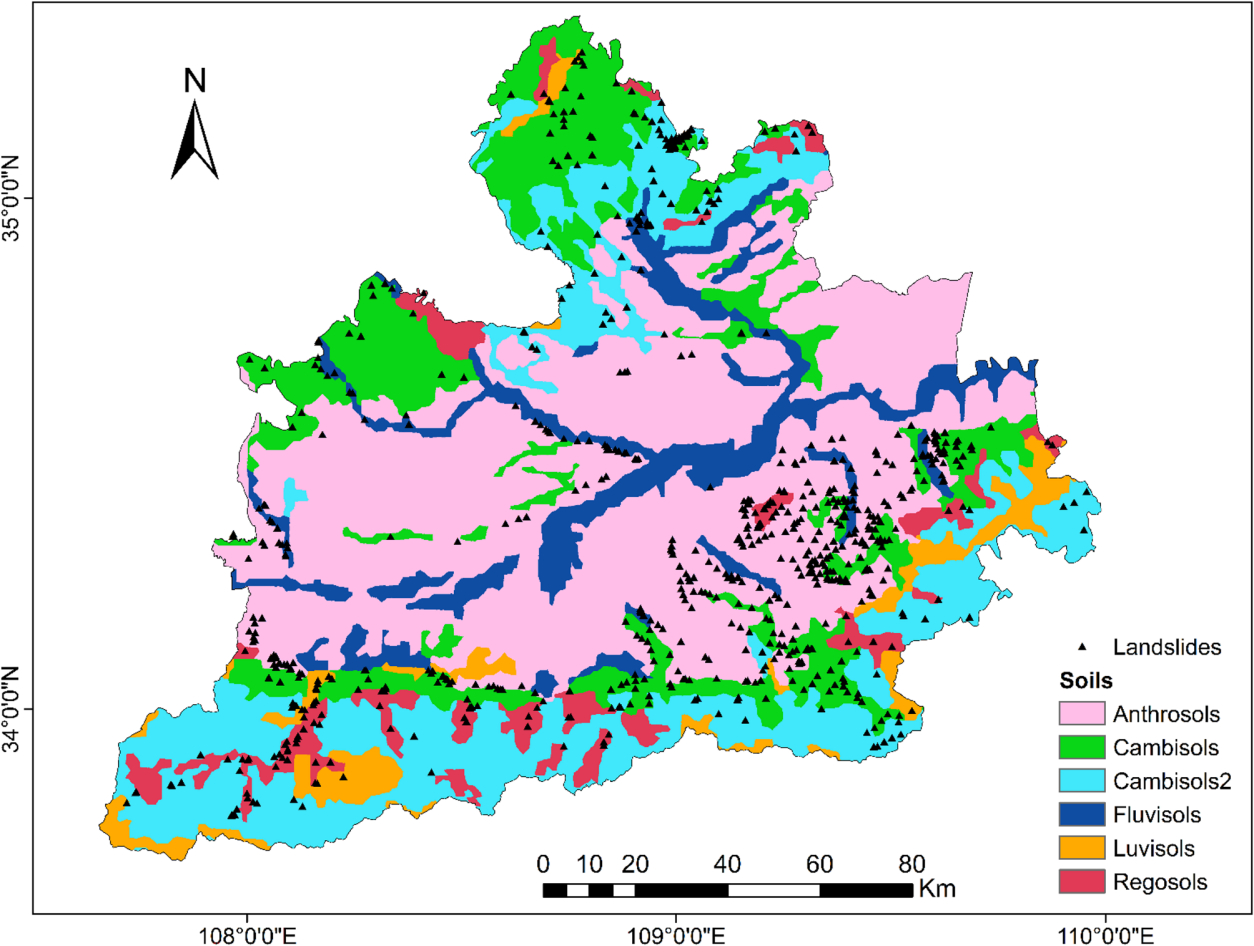
Interdependence of the Great Xi’an Region landslides with the soil map.

As shown in Table 4 and Fig. 11, about 35% of the landslides are located on Anthrosols, 43% on cambisols, 9% on fluvisols, 10% on regosols, and the proportion of landslides on luvisols is the least, only 2%. Anthrosols and cambisols accounted for 78%. Combined with Figs. 10 and 11, it can be found that they are mostly located on the loess layer.

### Normalized weight for conditioning factors

The normalized weight values were calculated from the individual eigenvalues using the hierarchical analysis process in Section "Analytical hierarchical process (AHP)". Table 5 shows the derivation of the normalized weights for each theme, with a CR value of 0.050. Similarly, Table 4 shows the normalized weights of the different features for each theme, with all features having a CR value of less than 0.10, which means that they passed the consistency test.

### Landslide susceptibility assessment

**Figure 12:**
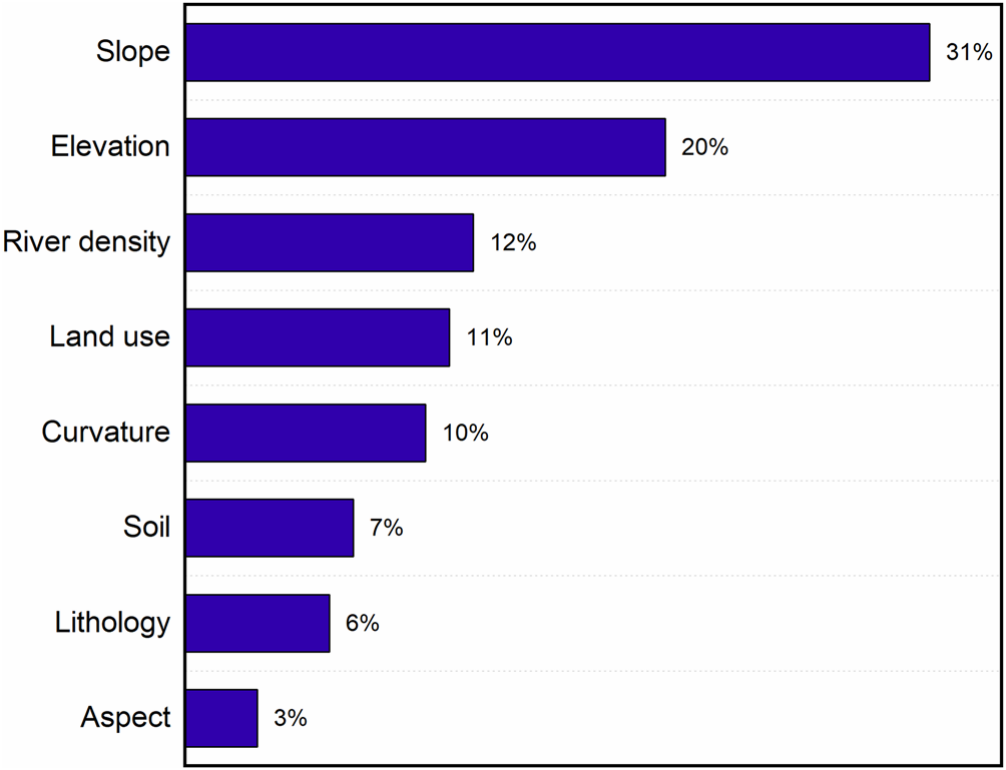
The relative importance of the different factors at the origin of the landslides according to the AHP approach.

To produce the landslide susceptibility map of the study area, it is necessary to comprehensively consider the cumulative effects under different landslide triggering factors. The order of weight calculated by the pairwise matrix (Table 5) of 8 landslide factors is shown in Fig. 12. They are: Slope (34%), Elevation (20%), River density (12%), Land use (11%), Curvature (10%), Soil (7%), Lithology (6%) and Aspect (3%).

**Figure 13:**
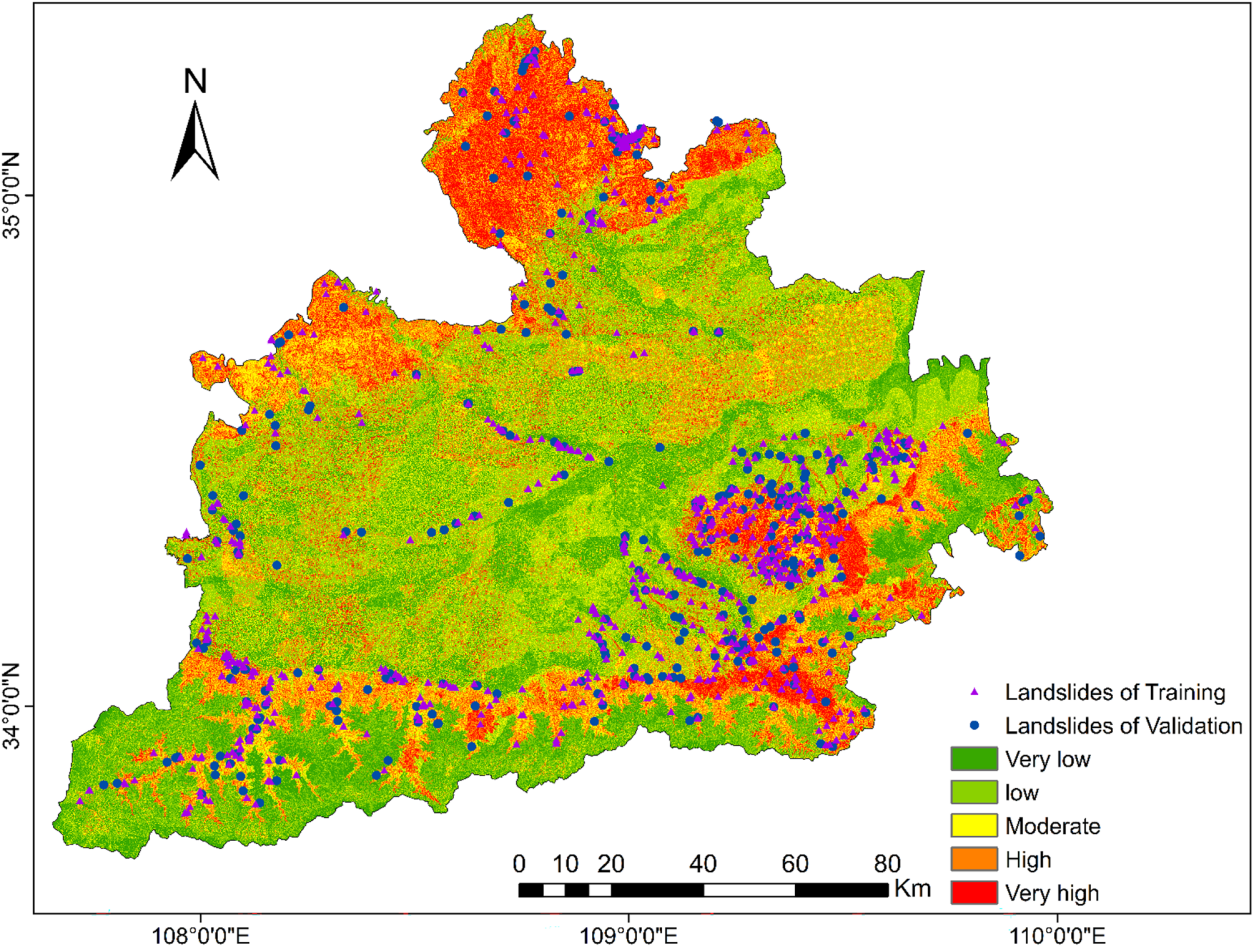
Landslide susceptibility map of Great Xi’an Region.

**Figure 14:**
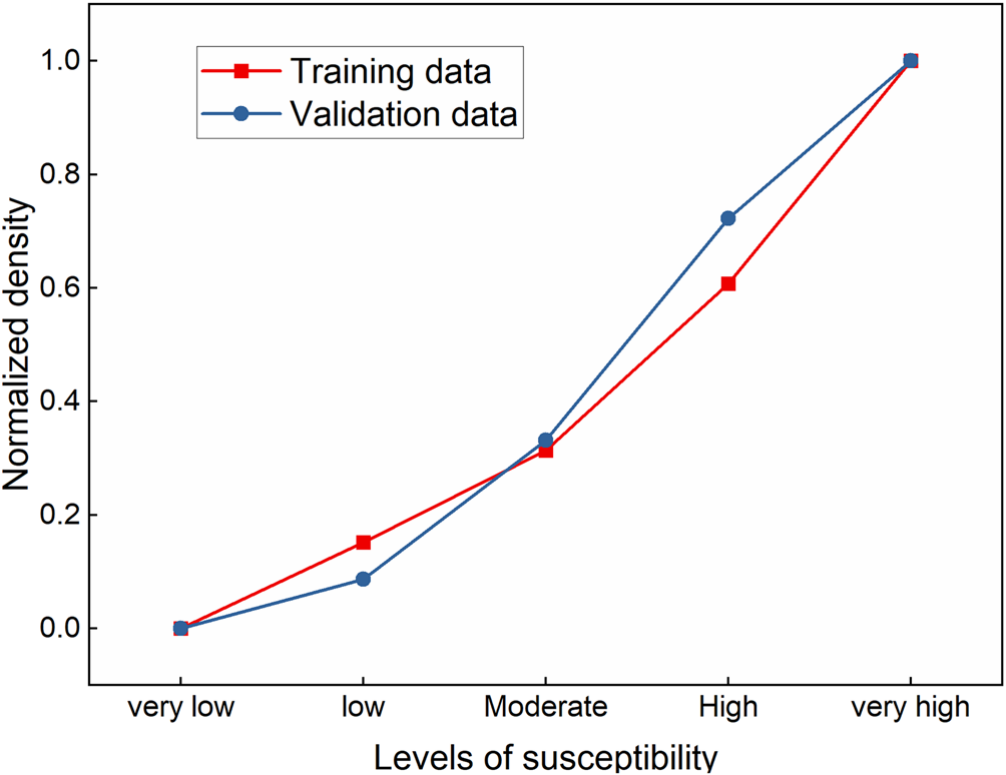
Distribution of landslide density after normalization.

The landslide susceptibility map (Fig. 13) is divided into five grades: very low, low, moderate, high, and very high, equivalent to 17.80%, 28.70%, 23.03%, 19.41% and 11.06% of the total area of the study area. Table 6 shows the distribution of landslide data in the landslide susceptibility map. Among the training data, 3.86% of the data fell into the extremely low susceptibility area, 16.11% fell into the low susceptibility area, 21.44% fell into the moderate susceptibility area, and 31.03% and 27.56% fell into the high susceptibility and extremely high susceptibility area. In the verification data, 4.66% and 12.73% of the data fell into the very low and low susceptibility areas, 22.05% fell into the moderate susceptibility areas, and 34.47% and 34.47% fell into the high and very high susceptibility areas. The distribution of the two sets of data is consistent. The ratio of the number of landslides to the area of the susceptibility classification group can reflect the landslide density of different susceptibility areas. After normalization, as shown in Fig. 14, the normalized density distribution of the test data and the verification data has a good consistency, and the size of the landslide density is positively correlated with the size of the susceptibility, indicating that the landslide susceptibility map drawn using the AHP calculation method is reasonable.

**Table 6:** Key information of LS map created by AHP.

Sub-regions	Areas (%)	Train (%)	Test (%)
Very low	17.80	3.86	4.66
Low	28.70	16.11	12.73
Moderate	23.03	21.44	22.05
High	19.41	31.03	34.47
Very high	11.06	27.56	26.09

## Discussion

### Causes of landslides

The Great Xi’an Region is located on the southern margin of the Loess Plateau. The landslide mechanism in this region can be analyzed in the context of previous research results on landslides in the loess area.

#### Human activity

Human activities are the main cause of catastrophic landslides in the loess area^53,54^. The excavation of the slope toe in engineering construction will cause stress concentration and uneven settlement of the filling foundation. The construction of new urban areas and supporting facilities have changed the original geomorphic environment and geological and hydrological structure in the area. The change in crustal stress and environmental conditions will induce the formation of landslide structures and the occurrence of landslides^7,8^.

Road networks and pipelines are important projects connecting cities. Linear projects such as railways and highways are mostly built on the second and third terraces of rivers^8^. Long-term infiltration of rivers, slope cutting and unloading during construction, large-scale mechanical vibration, and long-term cyclic loading of vehicles during use, etc., promote the sliding surface to connect and the ancient landslide to revive.

Loess has strong water sensitivity and an obvious softening effect on underwater action, which is an important content in loess research^55–58^. The large-scale irrigation in agricultural activities makes the groundwater level rise and the hydrogeological structure change, which provides support for the occurrence of landslides.

#### Geomorphology

Landslide disasters in the study area are mostly concentrated on the edge of the tableland and gully-intensive areas, and rivers pass through these areas. Surrounding the river are steep slopes that are easy to collapse. These loess slopes can remain stable under undisturbed conditions. However, under the action of rainfall or irrigation, collapsibility, disintegration, solubility, and softening are reflected^8,59,60^, and it is easy to form landslide groups.

### Factors controlling landslides

Landslide disasters in the planning area of the Great Xi’an Region can be triggered by factors such as slope, altitude, slope direction, curvature, land use, river density, soil, and lithology. The qualitative analysis and AHP process of these factors are carried out, and the landslide susceptibility map is drawn. Next, the influence of each factor and the reliability of the landslide susceptibility map are discussed.

In this study, 56% of the landslides were located in aerosols and bisols. The eolian rock (loess) accounts for 65% of the landslide data. 61% of the landslides are located in the middle altitude classification, equivalent to 806–3754 m. 69% of the landslides are located on grade 2, grade 3, and grade 4 slopes, that is, between 5 and 30°. The river density levels of 35–51 and 81–144 distributed 58% of the landslide data. Cultivated Land and Artificial surfaces land use types accounted for 67% of the landslide. It shows that the Great Xi’an Region is mainly a loess landslide and mainly concentrated in the middle altitude area, low, medium slope, river distribution dense human residential area or agricultural area. Therefore, natural factors such as topography hydrology and human activities have played a huge role in landslide disasters in the Great Xi’an Region. The results are similar to studies in other parts of the world, such as Sado Island in Japan, the Haraz watershed in Iran, Huizhou in China, and the Bafoussam-Dschang region in West Cameroon^33,61–63^.

In the study of profile curvature, the distribution of landslides on convex, concave, and straight is 32%, 37%, and 31%. The study of the slope aspect shows that the distribution of landslides on the plane is the least (3%), followed by the northwest (8%), and the other slope aspects are scattered (11–14%). The results show that curvature and aspect have limited influence on landslides in this area. Landslide studies in other regions have similar conclusions^33,64^.

Although studies have shown that human activities are the main factor triggering loess landslides, it should also be noted that natural factors such as slope, elevation, and rivers provide the conditions for landslides to occur, and thus they contribute significantly to landslide susceptibility. In addition, during human engineering activities, such as roads and tunnels, unstable slopes along the route are often reinforced, and these measures change the original topography and hydrological conditions of the area, thus making landslide susceptibility lower. Since landslide sensitivity maps are used in urban planning and disaster prevention, they should focus on areas of high landslide susceptibility created by natural factors, which will facilitate subsequent investigation and treatment.

### Reliability of landslide susceptibility map

Quantify landslide factors using the AHP method and draw a landslide susceptibility map (Fig. 13) of the Great Xi’an Region, which can reflect the combination of interactions and their effects.

The surface of the whole Great Xi’an Region is divided into five landslide-prone areas: Very low (17.8%), Low (28.7%), Moderate (23.03%), High (19.41%), and Very high (11.06%). Most of the plain areas are located in extremely low and low prone areas, while the tableland and intermountain gullies are located in high and extremely high prone areas, indicating that slope and altitude play a leading role in classification. This is consistent with most previous studies on landslide susceptibility^33,65,66^.

Therefore, in the study area, the slope provides a morphological basis for the occurrence of landslides. Altitude has obvious effects on human activities, vegetation types, and water resource distribution. These two factors are the basis of landslide elements, and it is difficult to directly improve them in actual landslide prevention and control. Therefore, it is necessary to pay attention to other control factors. River density (12%), human activities (11%), and curvature (10%) also contribute greatly to landslides in this area, which is consistent with the influencing factors of loess landslides and can be used as a basis for judging landslide susceptibility. Soil (7%), lithology (6%), and slope aspect (3%) have a small impact on the landslide susceptibility in this area. It may be difficult to judge the landslide susceptibility in the study area from this aspect.

In general, the hilly areas in the south and north of the Greater Xi’an region are high-susceptibility zones, and these areas are interspersed with gullies and rivers of various sizes, which provide a natural basis for the occurrence of landslides. The central of the study area is the Weihe Plain, which is flat, but there are many loess tablelands, such as the Bailu tableland^67^ and south Jingyang platform^68^. A large amount of cultivated land is distributed on these tablelands, and landslides occur from time to time due to the infiltration of agricultural irrigation water, so these loess tablelands are also high-susceptibility areas.

### Contributions and limitations of this study

The Loess Plateau is one of the five places in China most prone to landslides^69^. Tableland, beam, and hill are the main landforms of the Loess Plateau^70^. Some of these large cities are situated in the tableland and hilly areas of the Loess Plateau. According to statistics^71^, loess tablelands are mainly located in the northern part of Xi’an, the eastern part of Lanzhou, and the area around Taiyuan, and loess hills are mainly located in the western part of Taiyuan, the northern part of Xi’an, and the area around Lanzhou. In addition, studies have shown that landslides in these same geomorphic areas appear to have similar landslide mechanisms, such as the BaiLu Tableland in Xi’an City^72^ and the Heifangtai Tableland in Gansu Province^73^, both of which are dominated by arable land on the tableland surface, and large-scale landslides have occurred under the long-term infiltration of water, resulting in serious human casualties and economic losses. In the past, the study of loess landslides mainly focused on the mechanism of loess mechanical properties on loess landslides, prevention and control, and factors affecting them^8^, while relatively few studies have been conducted on the distribution of landslide susceptibility in large regions. This study investigates the landslide susceptibility zoning in the Greater Xi’an Planning Area and explores the macro-control factors of landslides in this area. Due to the similarity of topography and landslide mechanisms in loess landslide-prone areas, this study can provide a reference for the investigation and study of landslides during the expansion and construction of other cities in the Loess Plateau, such as Lanzhou and Taiyuan cities.

It should be noted that although the results of this study are in good agreement with the distribution of historical landslides, there are still some limitations in this study. The hierarchical analysis method (AHP) is a traditional method of landslide susceptibility zoning, and the greatest advantage of this method is that it does not require accurate historical landslide data^25,26^, which is why it is still widely used. However, it must be recognized that machine learning methods based on historical landslide inventories are developing more rapidly in this field, and it is significant to compare the landslide susceptibility prediction performance of different models^74^. Therefore, more accurate landslide databases, such as event-based and time-based landslide inventories, should be established in the future to explore the optimal landslide susceptibility model for each region of the Loess Plateau by comparing the applicability of various models.

## Conclusion

Based on the AHP method, the landslide susceptibility map of the Great Xi’an Region was established. The results show that slope, altitude, river density, land use, curvature, soil, lithology, and slope aspect trigger landslides. In the landslide disaster in the Great Xi’an Region District, terrain and hydrological conditions such as altitude, slope, and river provide the basis, and construction land and agricultural activities are the main inducing factors of human activities. The five-level landslide susceptibility map drawn by comprehensive trigger factors shows that the edge of the loess tableland, the gully area with dense rivers, and the edge of the entire Weihe Plain are the landslide-prone areas, which should be paid attention to in engineering construction and agricultural irrigation. The landslide susceptibility map can be used for planning and disaster prevention in the greater Xi’an region. The output of the study can support landslide studies at similar sites on the Loess Plateau.

### Supplementary Information

Supplementary Information.

## Data Availability

All data generated or analysed during this study are included in this published article [and its supplementary information files].
