# Supplementary figures and images for: Landslide susceptibility zonation using the analytical hierarchy process (AHP) in the Great Xi’an Region, China
Source: Sci Rep. 2024 Feb 5;14:2941. doi: 10.1038/s41598-024-53630-y (PMC10844316; doi:10.1038/s41598-024-53630-y)

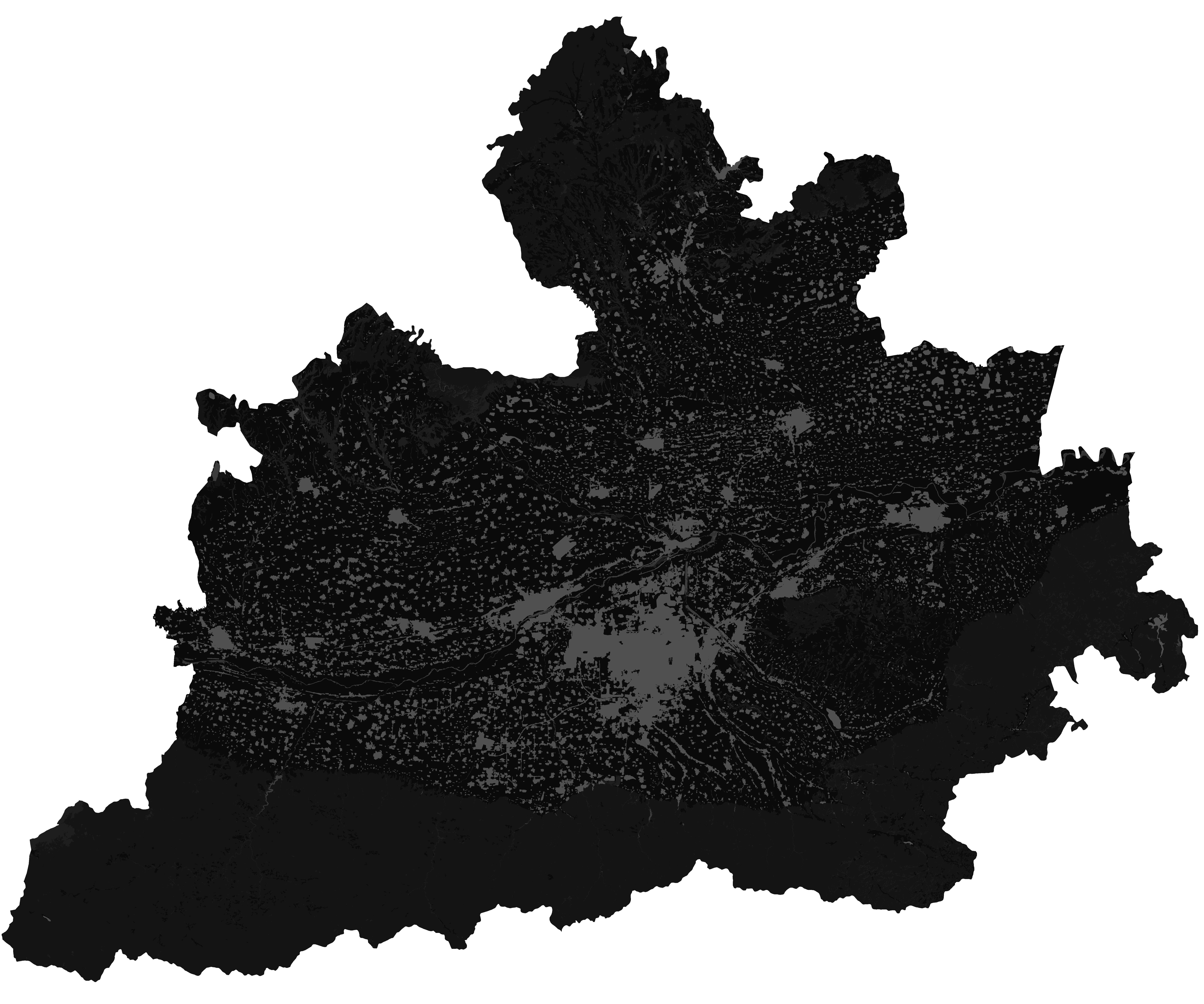

Supplement: Supplementary file 1 — Supplementary Information. [file 41598_2024_53630_MOESM1_ESM.zip › Data/landuse.tif]

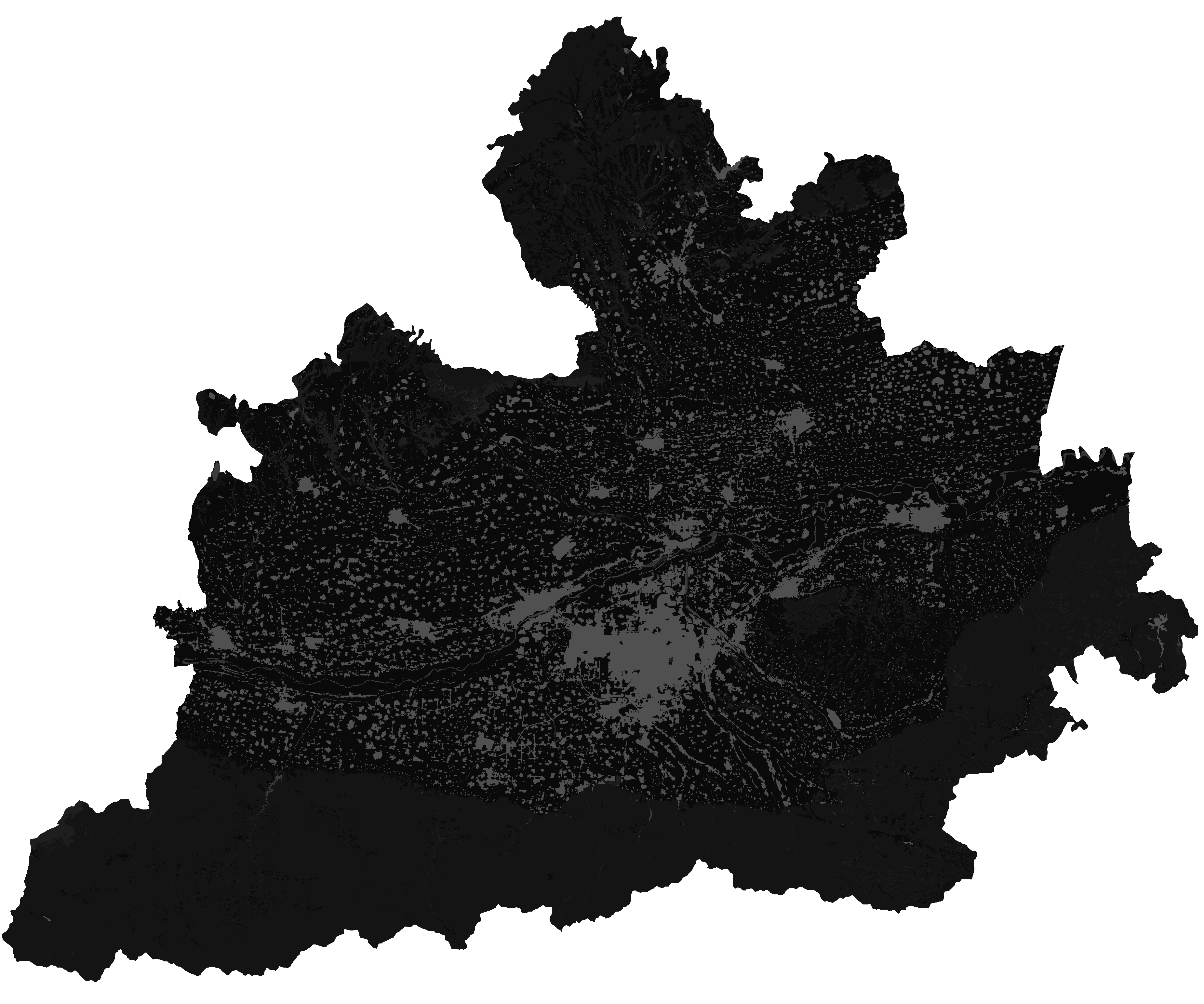

Supplement: Supplementary file 1 — Supplementary Information. [file 41598_2024_53630_MOESM1_ESM.zip › Data/landuse.tif.ovr]
